# Biochemometry-Based Discovery of Phenylpropanoids from *Azadirachta indica* Fruits as Inhibitors of In Vitro Osteoclast Formation

**DOI:** 10.3390/molecules27113611

**Published:** 2022-06-04

**Authors:** Ammar Tahir, Carina Kampleitner, Theresa Wirglauer, Ulrike Grienke, Oskar Hoffmann, Judith M. Rollinger

**Affiliations:** 1 Division of Pharmacognosy, Department of Pharmaceutical Sciences, University of Vienna, Josef-Holaubek-Platz 2, 1090 Vienna, Austria; ammar.tahir@univie.ac.at (A.T.); ulrike.grienke@univie.ac.at (U.G.); 2 Division of Pharmacology and Toxicology, Department of Pharmaceutical Sciences, University of Vienna, Josef-Holaubek-Platz 2, 1090 Vienna, Austria; carina.kampleitner@univie.ac.at (C.K.); a1202312@unet.univie.ac.at (T.W.); oskar.hoffmann@univie.ac.at (O.H.)

**Keywords:** *Azadirachta indica*, phenylpropanoids, biochemometry, osteoporosis, inhibition of osteoclast differentiation

## Abstract

(1) Background: Inhibition of osteoclast differentiation is the key approach in treating osteoporosis. However, using state-of-the-art treatments such as bisphosphonates and estrogen-based therapy is usually accompanied by many side effects. As opposed to this, the use of natural products as an osteoporotic remedy delivers promising outcomes with minimal side effects. (2) Methods: In the present study, we implemented a biochemometric workflow comprising (i) chemometric approaches using NMR and mass spectrometry and (ii) cell biological approaches using an osteoclast cytochemical marker (TRAP). The workflow serves as a screening tool to pursue potential in vitro osteoclast inhibitors. (3) Results: The workflow allowed for the selective isolation of two phenylpropanoids (coniferyl alcohol and sinapyl alcohol) from the fruits of neem tree (*Azadirachta indica*). These two isolated phenylpropanoids showed a very promising dose-dependent inhibition of osteoclast differentiation with negligible effects in terms of cell viability. (4) Conclusion: The presented workflow is an effective tool in the discovery of potential candidates for osteoclast inhibition from complex extracts. The used biochemometric approach saves time, effort and costs while delivering precise hints to selectively isolate bioactive constituents.

## 1. Introduction

Bone diseases concern a wide population of individuals, both men and women, and affect their life quality. Furthermore, they project a huge burden on society and the health system. These diseases are profoundly reliant on bone metabolism, which is mainly regulated through the communication between bone-forming osteoblasts (OB) and bone-resorbing osteoclasts (OC) [1]. Thus, osteoporosis usually occurs when this dynamic homeostasis is disturbed by an increased OC activity [1,2,3]. 

OC originate from hematopoietic stem cells and differentiate into specialized multinucleated giant cells by cell fusion of mononuclear precursor cells capable of resorbing the bone. Upon differentiation, OC display certain markers that can be used to evaluate differentiation and resorbing activity. Assessing the differentiation and activity of OC is hence considered vital in studies evaluating existing and potential new therapies. In addition to descriptive morphology (cellular size, shape and number of nuclei), OC can be identified via more precise and selective hallmarks, i.e., the presence and secretion of tartrate-resistant acid phosphatase (TRAP) and cathepsin K, actin ring formation, vitronectin receptor or resorption ability [2,4,5]. 

TRAP is a glycosylated monomeric enzyme localized in lysosomes, vesicles, Golgi cisternae and in the ruffled border of OC precursors and mature OC. Its detection via histochemical staining or activity assays is an established and commonly used marker for identifying OC and studying their effects on osteoclastogenesis [4]. 

By inhibiting the differentiation of OC precursors and the bone-resorbing activity of mature OC, many bone diseases can be remedied. Bisphosphonates, for instance, are the most commonly used drugs to inhibit OC activity. They attach to hydroxyapatite binding sites, particularly on surfaces enduring active resorption [5] and inhibit the mevalonate pathway. However, they are associated with short- and long-term adverse effects, among them the osteonecrosis of the jaw [6]. Additionally, estrogen-based therapies were used to counteract osteoporosis [7] but were also endowed with side effects [8]. Later developed treatment modalities target specific cytokines or glycoproteins. One of these drugs is the RANKL inhibitor denosumab, which prevents RANKL-induced differentiation and activation of OC, thus inhibiting bone resorption in vivo.

On the other hand, the use of natural products has been reported to be effective in the treatment of osteoporosis due to their long-term safe use and cost factors [9,10]. Many formulations in traditional Chinese medicine have been applied successfully to increase bone mineral density and improve the life quality of patients [11]. Several compounds derived from natural sources such as curcumin [12], berberine [13] and tanshinone [14] have been reported to be effective in the inhibition of OC activity in vitro. 

*Azadirachta indica* A. Juss. is a medicinal plant belonging to the Meliaceae family and is native to the Indian subcontinent. It is commonly known as neem tree, miracle tree or the “pharmacy of the village” tree [15]. For centuries, different parts of this tree have been used intensively in traditional medicine [16,17]. Extracts of the different neem tree organs comprise a diversity of chemistries such as flavonoids, saponins, tannins, limonoids, gallic acid, di- and triterpenoids, and coumarins [18,19]. These constituents contribute to a broad spectrum of activity, including anti-inflammatory, antiarthritic, antipyretic, antimicrobial, antitumor and immunomodulatory, as previously summarized by Biswas et al. [20]. Neem tree metabolites have been reported to modulate numerous signaling pathways at the molecular level, as reviewed by Gupta et al. [21]. 

To search for new strategies for identifying anti-osteoporotic drug candidates from natural sources, an extract screening was performed. Within this screening, a methanolic extract of the fruits of *A. indica* was identified as the most promising plant source containing constituents that were able to reduce OC differentiation in vitro. Our aim was to unravel which constituents of the extract are responsible for the observed activity. For this purpose, the recently established biochemometric workflow ELINA (Eliciting Nature’s Activities) was applied [22,23]. With this approach, the two phenylpropanoids coniferyl alcohol (CA) and sinapyl alcohol (SA) were identified to contribute significantly to the OC inhibitory activity of the extract tested. 

To our knowledge, this is the first report describing the potential inhibitory activity of CA and SA isolated from the fruits of *A. indica* on OC. This was achieved by applying a biochemometry-based discovery approach in combination with cell biological methods to estimate the inhibitory effects of the two compounds on OC differentiation.

## 2. Results

### 2.1. Extract Screening

**Figure 1:**
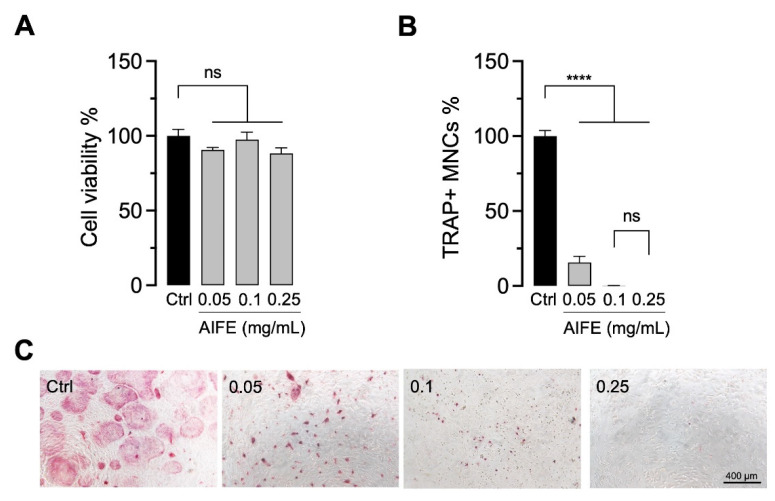
Natural product screening of *A. indica* fruit extract (AIFE). Primary mouse OB and bone marrow-derived OC precursors were cultured in OC differentiation medium and incubated with AIFE extract (0.05–0.25 mg/mL). Cell viability (**A**) day 3 and OC differentiation (**B**) day 5 was assessed, respectively. Relative cell viability was normalized to the vehicle control (ctrl, OC differentiation medium containing 0.1% DMSO) and is presented as percentage (mean ± SEM; *n* = 6). To evaluate osteoclastogenesis, cells were stained for TRAP. Graph demonstrates the quantification of TRAP+ stained multinucleated cells (TRAP+ MNCs) normalized to the vehicle control (ctrl) (mean ± SEM; *n* = 6). (**C**) Representative images illustrate TRAP+ OC morphology (pink cells) from small, less nucleated TRAP+ cells (in AIFE 0.05 and 0.1 mg/mL) to giant multinucleated OC (in the control group). *p* = ns; **** *p* < 0.0001.

For a natural product extract screening on potential OC inhibitory effects, OB and bone marrow OC precursors were co-cultured and incubated with *A. indica* fruits (AIFE) extract at different concentrations (0.05–0.25 mg/mL) and then assessed for their biocompatibility (at day 3) and effect on OC development (at day 5). In this course, the methanolic extracts of AIFE showed the most promising results in terms of cell viability (no cytotoxicity) and inhibition of OC formation. We found that osteoclastogenesis was inhibited without affecting cell viability. The OC morphology differed between the vehicle control (ctrl) and the AIFE-treated cultures. The presence of AIFE resulted in a significant reduction (*p* < 0.0001) of TRAP+ multinucleated OC (TRAP+ MNCs), less nucleation and smaller cell size. These observations were directly linked to the administered AIFE dosage (Figure 1). 

### 2.2. Biochemometry to Track Bioactive Constituents

**Figure 2:**
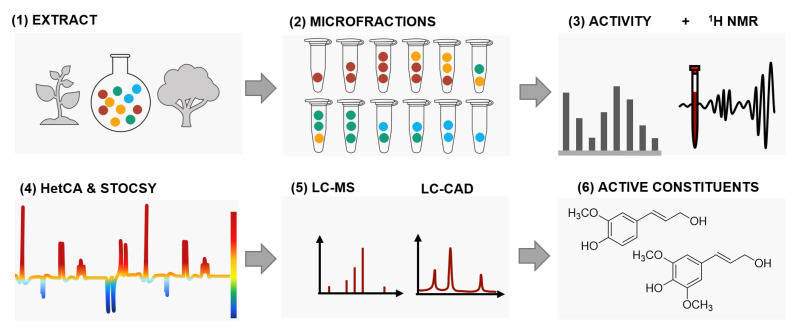
Schematic overview of the biochemometric workflow ELINA. A bioactive extract is fractionated to create a quantitative variance of constituents over microfractions. Aliquots of these fractions are forwarded to ^1^H NMR and bioactivity testing. The obtained ^1^H NMR data are statistically correlated with activity. Based on spectroscopic regions highlighted in red and blue, relevant structural features for activity (red) can be distinguished from inactive ones (blue). Additional implementation of LC-MS data and LC-CAD data further enables a straightforward identification and isolation of bioactive compounds.

To unravel the compounds responsible for the pronounced OC inhibition of the extract, the biochemometric approach ELINA (Grienke, 2019; Zwirchmayr, 2020) was applied. The acronym ELINA stands for “Eliciting Nature’s Activities” and is based on the correlation of bioactivity data and chemical data (e.g., ^1^H NMR) via multivariate statistics (e.g., heterocovariance analysis (HetCA), statistical total correlation spectroscopy (STOCSY)). A major advantage of this approach compared to, e.g., classic bioactivity-guided fractionation is the identification of bioactive compounds prior to isolation (Figure 2).

As a first step, an up-scaled methanolic fruit extract was fractionated using RP flash chromatography into 42 microfractions. The goal was to simplify and thus expand the structural complexity of the bioactive crude extract by the generation of microfractions with quantitative variances of constituents over several consecutive fractions. Aliquots of these 42 microfractions were equally prepared for (i) ^1^H NMR analyses to obtain quantitative and qualitative information on structural features, (ii) LC-MS-CAD (Liquid Chromatography coupled to Mass Spectrometry and Charged Aerosol Detector) investigations for semi-quantitative information and dereplication of constituents present in each microfraction in both, positive and negative mode, and (iii) bioactivity testing (c = 0.1 mg/mL).

**Figure 3:**
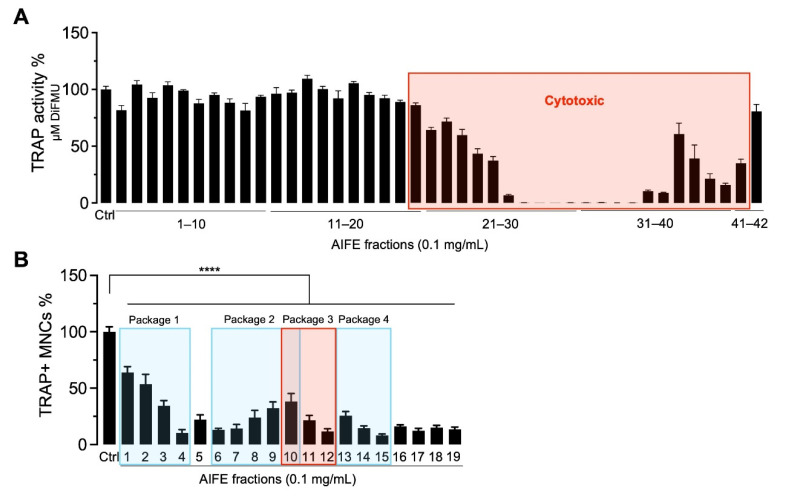
Effect microfractions of *A. indica* fruit extract (AIFE) on osteoclastogenesis. (**A**) OC related TRAP activity was quantified in cell lysates of co-cultures treated with AIFE microfractions 1 to 42 after 5 days of incubation. Intracellular TRAP enzyme activity was normalized to the vehicle control (ctrl) and is presented as percentage (TRAP activity%, mean ± SEM; *n* = 6). Microfractions 20–41 revealed cytotoxic effects and were excluded from further testing. (**B**) To correlate TRAP activity to numbers of multinucleated OC, AIFE microfractions 1–19 were further evaluated. Graph demonstrates the endpoint analysis of TRAP+ multinucleated cells (TRAP+ MNCs%, mean ± SEM; *n* = 6) enumerated under a light microscope. Statistical analysis showed a significant reduction of OC numbers for all microfractions (**** *p* < 0.0001). Packages were selected according to ascending or descending OC formation and were traced back quantitatively via the ^1^H NMR signals using HetCA data.

To upscale the bioactivity testing of these 42 AIFE microfractions, we firstly used a fluorescence-based approach to determine TRAP activity in cell lysates. The incubation of OB-OC co-cultures with the microfractions 20–42 led to cytotoxic effects with the detachment of cells and cell reduction and lack of TRAP, thus leading to the exclusion of these fractions in all further experiments (Figure 3A). To correlate TRAP activity measured in cell lysates to the number of multinucleated TRAP+ OC, non-toxic microfractions 1–19 were screened using the TRAP staining/counting assay (Figure 3B). Microfractions 4, 6, 12 and 15 were revealed to be the most promising samples, with 89.7%, 86.8%, 88.4% and 92.0% inhibition of OC formation, respectively.

**Figure 4:**
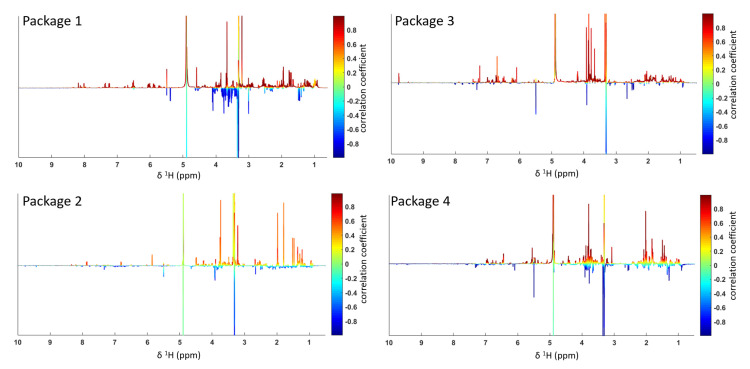
HetCA plots resulting from the covariance of ^1^H NMR data with TRAP data (as shown in Figure 3B).

The TRAP staining and counting assay revealed a variety of different potencies over the course of all 19 tested microfractions. Both decreasingly as well as increasingly active groups or so-called packages of fractions were observed. To perform the biochemometric ELINA approach, recorded ^1^H NMR data of the microfractions of a selected package were correlated with the bioactivity data by using the multivariate statistical tool heterocovariance analysis (HetCA) as described before (Grienke, 2019). HetCA plots were generated for all packages, as shown in Figure 4.

In total, four packages of three to five consecutive microfractions with a variance in activity were selected and are depicted as HetCA plots (Figure 3). The workflow (see Figure S1) to assess the priority of the packages comprised (1) an evaluation of HetCA plots to check for the strength of the correlation of ^1^H NMR data with TRAP data, followed by (2) a dereplication of the most bioactive fraction to screen for known compounds. The workflow resulted in the following findings:

1. The HetCA plot of package 1 shows signals with a strong correlation of ^1^H NMR data with TRAP data (red signals); dereplication showed that the main compound in Fr 4 with MW = 452 Da matches the mass of many neem specific tetranortriterpenoids, limonoids (such as nimonol, 1,2-dehydromeldenin, 23-deoxyazadironolide and 1,2-dehydromeldenin). Limonoids are already well known to have anti-osteoporotic activity [24,25,26]. Consequently, microfractions of this package were excluded from further isolation steps.
2. The HetCA plot of package 2 displays signals with a weak correlation of ^1^H NMR data with TRAP data; thus, this package was excluded from further steps.
3. The HetCA plot of package 3 demonstrates signals with a strong correlation of ^1^H NMR data with TRAP data. Since no MS/MS-based annotation to main features of known *A. indica* constituents was achieved for the signals obtained in Fr12, thus assuming novel metabolites, we focused on this package for further isolation steps.
4. The HetCA plot of package 4 shows a strong correlation of ^1^H NMR data with TRAP data. The dereplication showed that the main compound in Fr 15 with an MW of 720 Da matches the mass of a neem-specific triterpenoid, namely the limonoid azadirachtin, which has already been reported with anti-osteoporotic activity [27,28]. Consequently, this package was excluded from further steps.

### 2.3. Phytochemical Workup of Package 3

**Figure 5:**
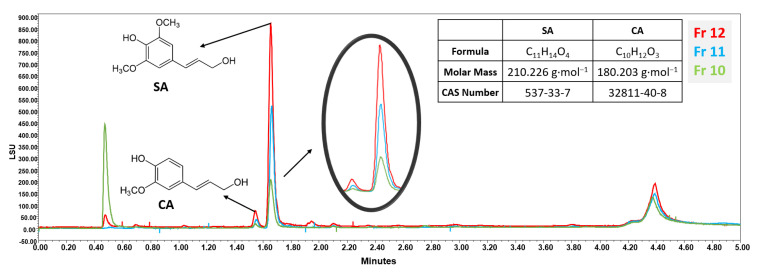
Two peaks (SA and CA) only correlate with TRAP assay data; these two peaks were later isolated using semi-prep-SFC. Peaks Rt 1.5 “CA” and Rt 1.7 “SA” are considered correlating because the concentrations match the bioactivity in Figure 3B. Peaks at Rt 0.5 min and Rt 4.4 min do not show this matching and thus were excluded from further isolation procedures.

Using package 3 including microfractions 10–12, a semi-preparative SFC (Supercritical Fluid Chromatography) chromatography method was developed. Firstly, the fractions of this package were analyzed with an analytical UHPSFC instrument using ELSD (Evaporative Light Scattering Detector) and UV detection. In addition to the HetCA plot, the chromatographic analysis revealed which peaks correlate with the bioactivity and are worth isolating (Figure 5).

**Figure 6:**
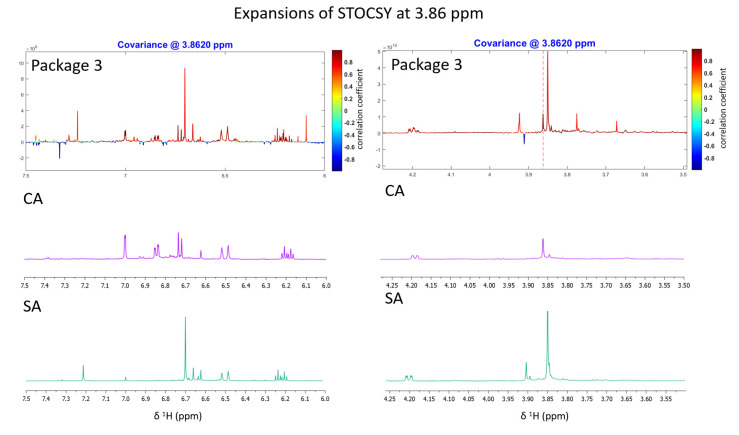
Matching of statistical total correlation spectroscopy (STOCSY) plot signals with ^1^H NMR signals of both isolated compounds (CA and SA). The signal at *δ*_H_ 3.86 was chosen to identify which molecule(s) share this “hot” feature. The plot is color-coded based on the correlation coefficient: blue = signals belonging to molecule(s) that do not have the signal at *δ*_H_ 3.86; red = signals belonging to molecule(s) that have the signal at *δ*_H_ 3.86. Left (6–7 ppm aromatic region); right (3.6–4.5 ppm).

The relevant fractions were subjected to semi-preparative SFC separation (Figure S2), which resulted in the isolation of two compounds. The structures of the two compounds, i.e., SA (0.74 mg) and CA (0.6 mg), were elucidated using 1D and 2D NMR spectroscopy and comparison with literature data [29,30]. We verified the soundness of our approach by comparing the NMR signals of the isolated constituents with the HetCA as well as STOCSY correlation signals of package 3 (Figure 6). One can undoubtedly see that the signals of the isolated constituents do match the red “high correlation” signals in the pseudospectrum. CA shows a slightly stronger positive correlation with activity than SA. 

### 2.4. Bioactivity of CA and SA

**Figure 7:**
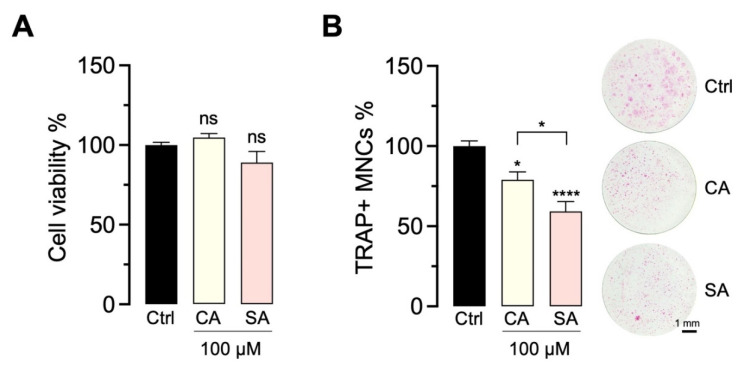
Bioactivity of CA and SA. The effect of isolated CA and SA (100 µM) on cell viability (**A**) day 3 and OC differentiation (**B**) day 5 was tested on co-cultures of primary mouse OB and bone marrow-derived OC precursors. Cell viability (mean ± SEM; *n* = 6) and numbers of TRAP+ multinucleated cells (TRAP+ MNCs, mean ± SEM; *n* = 9) were normalized to vehicle control (ctrl, OC differentiation medium containing 0.1% DMSO) and are presented as percentages. Representative images of TRAP+ MNCs demonstrate OC morphology and size (pink cells). *p* = ns; * *p* < 0.05; **** *p* < 0.0001.

The effect of CA and SA on OC viability and differentiation was tested at 100 µM and compared to the vehicle control. After staining for TRAP+ MNCs, we observed a significant inhibition of OC, while the two compounds did not change cell viability (Figure 7).

**Figure 8:**
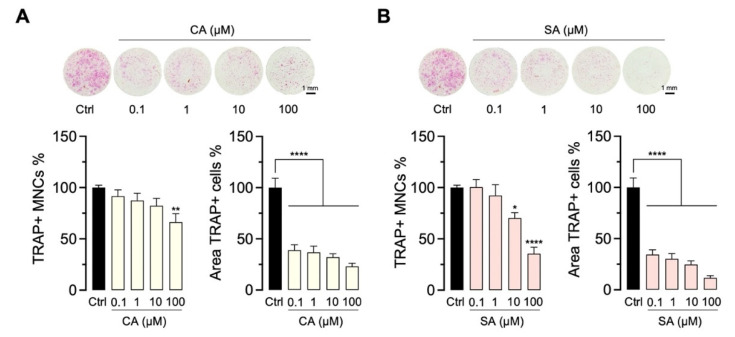
Dose–response testing of CA and SA for inhibition of OC formation. OC differentiation affected by CA (**A**) and SA (**B**) (0.1–100 µM) was investigated after treating the mouse OB-OC co-cultures for 5 days. OC numbers were evaluated in TRAP-stained cultures (TRAP+ MNCs, mean ± SEM; *n* = 9). Representative images of the TRAP staining are illustrated above the graphs. To quantify the observed changes in OC morphology (size), the area covered by TRAP+ cells was calculated (area TRAP+ cells, mean ± SEM; *n* = 9). Both parameters were normalized to vehicle control (ctrl, OC differentiation medium containing 0.1% DMSO) and are presented as percentages. * *p* < 0.05; ** *p* < 0.01; **** *p* < 0.0001.

For further dose–response experiments, commercially acquired CA and SA were tested for their effects on OC differentiation after 5 days of culture. CA (Figure 8A) and SA (Figure 8B) were able to inhibit the number (TRAP+ MNCs) of OC dose-dependently between 0.1 and 100 µM. These results are in agreement with the findings obtained with SA and CA isolated from the *A. indica*. In addition, treated co-cultures were analyzed for OC size changes. We observed that the treatment groups showed a significantly smaller area of TRAP+ cells than untreated controls.

## 3. Discussion

In this work, we selectively isolated two anti-osteoporotic phenylpropanoids from a defatted methanolic extract of neem tree fruits. The structure class of phenylpropanoids belongs to the family of phenylalanine-derived natural products. They are hydroxycinnamyl alcohol monomers that form building blocks of many important bioactive phytochemicals. Although there are many reports on the anti-osteoporotic activity of phenylpropanoids [31], investigations on the molecular level are limited [32,33,34]. Some phenylpropanoids possess the capacity to positively modulate the metabolism of OB. For example, dehydrodiconiferyl alcohol was previously reported to promote bone morphogenetic protein 2 (BMP-2)-induced osteoblastogenesis with no cytotoxic effects [35]. Even derivatives of phenylpropanoids, such as the lignin pinoresinol glucoside, have been shown to possess these modulatory effects and to exert an anabolic effect on the skeleton through the modulation of OB differentiation [36]. 

In the presented study, we observed a dose-dependent inhibitory effect of the crude AIFE extract and the two phenylpropanoids on OC differentiation without affecting cell viability. Thereby, our results confirm previous studies reporting similar phenylpropanoids to possess modulatory effects on bone metabolism; for instance, CA and hydroxycinnamic acid isolated from *Sambucus sieboldiana* were reported to have an inhibitory effect on the parathyroid hormone (PTH)-stimulated bone resorption at 20 and 200 µM [37]. Syringin (sinapyl alcohol 4-*O*-glucoside) and coniferin (coniferyl alcohol β-D-glucoside) were reported to inhibit osteoclastogenesis in ovariectomized mice and to show anti-osteoporotic activities [38,39].

In this study, it is the first time that a biochemometric approach was applied for the identification and isolation of OC inhibiting phenylpropanoids form a complex mixture. We utilized an established cytochemical marker (TRAP) for the identification of OCs, which offers a better way of characterizing them in terms of count and size in comparison to traditional methods [40]. On the other hand, and in contrast to a bioactivity-guided isolation procedure, the applied biochemometric approach ELINA showed to have many advantages: 

The implementation of bioactivity-oriented packaging enabled us to decide upon packages worth going for further phytochemical workup on fractions containing compounds with not yet described bioactivities. While others, after LC-MS/MS dereplication, have been excluded from further isolation steps due to known bioactive constituents. This targeted approach saves time, costs and effort in comparison to a bioassay-guided isolation procedure. 

Even in the case of a successful dereplication as in packages 1 and 4, ELINA provided a proof of concept since it was able to uncover previously reported bioactivities of these identified chemistries from complex mixtures. 

The robustness of ELINA allowed identifying different bioactive compound classes, which in total may contribute to the overall bioactivity of the extract. 

However, the approach also has its limitations. As seen in Package 3, a dereplication of SA and CA was not possible due to the dynamic range limitations of LC-MS. Phenylpropanoids are a very unstable species in ESI-MS and usually suffer insource fragmentation, thus failing to show the nominal mass in MS1 experiments. Accordingly, despite their abundance, they were not successfully dereplicated. For future biochemometric applications, this drawback could eventually be diminished by utilizing GC-MS for a dereplication in parallel to LC-MS.

In conclusion, the targeted biochemometric approach for the identification and isolation of the compounds inhibiting OC differentiation provides an efficient approach for the successful discovery of pharmacologically active compounds to modulate OC function. Based on the evaluated in vitro profile of SA and CA, they might be useful candidates to inhibit excessive bone resorption. However, further research is needed to gain a profound knowledge of their effects on OC, e.g., determining their influence on resorption, and to unravel molecular targets and mechanisms triggering impaired cell differentiation.

## 4. Materials and Methods

### 4.1. Plant Material, Chemicals and Reagents

Ground fruits of *A. indica* were provided by PADMA AG, Produktion/CH-8620 Wetzikon; Lot-Nummer 21357301. All solvents for extraction, flash chromatography, LC-MS and SFC were purchased from VWR Chemicals. Compressed 4.5 grade CO_2_ (purity ≥ 99.995%) was purchased from Messer. Coniferyl alcohol (C_10_H_12_O_3_, MW = 180.203 g mol^−1^) and sinapyl alcohol (C_11_H_14_O_4_, MW = 210.226 g mol^−1^) were bought by Sigma Aldrich (Merck, Darmstadt, Germany). 

### 4.2. Mouse co-Culture Model

To study the effect of the methanolic extract, microfractions and compounds on osteoclastogenesis, we used a primary mouse co-culture model of calvarial-derived neonatal OB, isolated by enzymatic digestion, and bone-marrow OC precursors as previously described [41,42]. OB (4.2–6.5 × 10^4^ cells per cm^2^) were seeded on tissue culture plastic in a culture medium containing ⍺MEM (Gibco, Thermo Fisher Scientific, Vienna, Austria), 10% heat-inactivated fetal bovine serum (FBS, Gibco) and 1% penicillin/streptomycin (Gibco). After 24 h, OC precursors harvested from femurs and tibiae of 8–12-week-old BALB/c mice were added to the OB, and cultures were cultivated in OC differentiation medium, basal culture medium supplemented with 1 nM 1,25-(OH)_2_)-vitamin D_3_ (Sigma Aldrich, Vienna, Austria) and 1 µM prostaglandin E2 (Cayman Chemicals, Hamburg, Germany), containing the substance of interest (extract, microfractions or compound) for either 3 or 5 days at 37 °C and 5% CO_2_. Extracts and compounds were evaluated for biocompatibility and OC differentiation by different assays. 

The naïve neonatal and adult BALB/c mice used for cell isolation were either bred in our in-house animal facility (Division of Pharmacology and Toxicology, University of Vienna, Austria) or purchased at Charles River Laboratories (Sulzfeld, Germany). Animals in our facility were housed in stable conditions with a 12 h dark/light cycle and ad libitum access to food (complete feed for mice, VR1126-000) and water.

### 4.3. Cell Viability and Osteoclast Formation

For cytotoxic profiling, we assessed cell viability using an MTS assay (CellTiter96^®^AQ_ueous_ One Solution Cell Proliferation Assay, Promega, Vienna, Austria) according to the manufacturer’s instruction after 3 days. Mouse OB-OC cultures were incubated directly with the MTS reagent for 1 h at 37 °C and 5% CO_2_. Metabolically active cells produce a colored, medium-soluble product. Its absorbance was recorded at 490 nm using a standard microplate reader (Infinite^®^ M200 Pro, Tecan, Männedorf, Switzerland). 

OC differentiation was studied by evaluating the presence of tartrate-resistant acid phosphatase (TRAP) either by histochemical staining or a spectrophotometric approach. Both assays make use of the enzymatic activity of TRAP by adding substrate solutions that are hydrolyzed enzymatically, resulting in an insoluble or fluorescent dye.

For TRAP staining, we followed a protocol that was previously reported in Kampleitner et al. [41]. Briefly, cells were fixed in 10% buffered formalin at room temperature on day 5 of co-culture and incubated with the TRAP staining solution at 37 °C for 10 min. TRAP+ multinucleated cells (TRAP+ MNCs) with 3 or more nuclei were considered as mature OC and enumerated under a light microscope (Nikon Diaphot 300, Tokyo, Japan). 

TRAP activity was measured in cell lysates (at day 5 of co-cultures) with the EnzChek^®^ Phosphatase Assay Kit (Invitrogen, Thermo Fisher Scientific) for pre-screening of AIFE microfractions. Therefore, the conditioned medium was removed, cells were washed once with phosphate-buffered saline (PBS, Gibco, Thermo Fisher Scientific) and plates were subsequently frozen at −80 °C. After 24 h, cultures were thawed, and cells were lysed using a cell lysis buffer (CyQuant^TM^ cell lysis buffer, Invitrogen, Thermo Fisher Scientific) at room temperature for 5 min on an orbital shaker. For TRAP activity measurements, cell lysates and substrate solution (200 µM 6,8-difluoro-4-methylumbelliferyl phosphate (DiFMUP) in reaction buffer pH 5.0) were mixed with a dilution ratio of 1:2 and incubated at 37 °C for 15 min. Fluorescence readings of the dephosphorylated substrate were taken on a standard microplate reader (excitation 358 nm; emission 455 nm) and compared to a standard curve of the unphosphorylated product (6,8-difluoro-7-hydroxy-4-methylcoumarin, DiFMU). 

In addition, changes in OC size were evaluated for co-cultures treated with commercially purchased compounds after TRAP staining. Images of TRAP+ stained cultures were loaded into Adobe Photoshop CC 2019 and thresholded to determine the relative area covered by TRAP+ stained cells. A schematic representation of the experimental design to study *A. indica* fruit extract, microfractions and compounds is shown in Supplementary Material (Figure S3).

### 4.4. Nominal Mass Dereplication Using LC-MS

LC-MS (liquid chromatography coupled to mass spectrometry) dereplication of bioactive fractions was performed using a Dionex UltiMate 3000 RLC instrument system (Thermo Fisher Scientific—San Jose, CA, USA) coupled to Thermo LTQ XL ion trap mass spectrometer (Thermo Fisher Scientific—San Jose, CA, USA) and an electrospray ionization source (ESI) probe. Dereplication was performed using a gradient run on a Phenomenex Kinetex-C_18_ column (2.1 × 100 mm, 1.7 µm). Solvent A: Water–acetic acid–formic acid (99.9:0.05:0.05); Solvent B: Acetonitril–acetic acid–formic acid (99.9:0.05:0.05). The following gradient was applied: 0–5 min: 0% B, 5–26 min: 95% B, 26–28 min 95% B, 28–30 min 0% B. Then, 5 µL of the dissolved sample (methanol–water 25:75) was injected. The nominal mass of the most intense features was compared with peer-reviewed literature on known *A. indica* compounds using the Chemical Abstracts Service (CAS) resource SciFinder, as a curated database of chemical and bibliographic information.

### 4.5. Crude Extract Generation and Flash Chromatography

In total, 990 g of crude plant material was defatted with 1.5 L n-hexane; the extract was collected in a round bottom flask and was evaporated under a vacuum. The rest of the material was then macerated with MeOH (500 mL at 22 °C for 24 h). For an exhaustive extraction, the procedure was repeated four times. The extract was collected in a round bottom flask and was evaporated under vacuum (methanol extract = 48.5 g). An aliquot (3.5 g) of the dried methanol extract was subjected to flash column chromatography (CC). Flash CC was performed on an Interchim puriFlash 4250 system (Montluçon, France), equipped with ELSD, PDA and a fraction collector, controlled by Interchim Software. A PuriFlash C_18_ HQ column (15 µm, 120 g) served as the stationary phase. The mobile phase consisted of water (A) and 20% MeOH in ACN (B) (flow rate, 34 mL/min). By applying a gradient (0’ 95% A/5% B, 10’ 95% A/5% B, 75’ 20% A/80% B, 80’ 5% A/95% B, 120’ 5% A/95% B) the extract was separated into 42 fractions (Fr1–Fr42). 

### 4.6. Semi-Preparative SFC

Package 3 was enriched 3 times using the Flash CC procedure explained in Section 4.5 above and then pooled together. The pooled fractions (70 mg) were subjected to semi-preparative supercritical fluid chromatography (SFC) and further resulted in the two isolated compounds. Semi-preparative SFC was performed on a Waters Prep-15 System (Waters, Milford, MA, USA) equipped with an evaporative light scattering detector (ELSD), a photodiode array (PDA) and a fraction collector. A Waters Viridis Prep BEH 2-EP column (5 µm; 10 × 250 mm) served as the stationary phase, and data were analyzed using MassLynx. The mobile phase consisted of supercritical CO_2_ (A) and ACN:MeOH as an organic modifier (B) (temperature, 40 °C; flow rate, 15 mL/min). The following gradient was used: 0’ 85% A/15% B, 3’ 85% A/15% B, 5’ 50% A/50% B, 7’ 50% A/50% B, 9’ 85% A/15% B, 10’ 85% A/15% B.

### 4.7. NMR Measurements

NMR experiments were performed by using a Bruker Avance 500 NMR spectrometer (UltraShield) (Bruker, MA, USA) with a 5 mm switchable probe (TCI Prodigy CryoProbe, 5 mm, triple resonance inverse detection probe head) with z-axis gradients and automatic tuning and matching accessory (Bruker BioSpin, Billerica, MA, USA). The samples (1 mg/mL) were measured at 298 K in fully deuterated methanol referenced to the residual non-deuterated solvent signals. The resonance frequency for ^1^H NMR was 500.13 MHz and for ^13^C NMR 125.75 MHz. Standard 1D and gradient-enhanced 2D experiments, such as double quantum filtered (DQF) COSY, NOESY, HSQC and HMBC, were used as supplied by the manufacturer.

### 4.8. Statistical Analysis

Statistical analysis was performed using one-way analysis of variance (ANOVA) with Tukey’s post hoc test for multiple comparison (GraphPad Prism 9, USA), and *p* < 0.05 was considered statistically significant. 

## Data Availability

Not applicable.

## Supplementary Materials

The following supporting information can be downloaded at: https://www.mdpi.com/article/10.3390/molecules27113611/s1, Figure S1: Biochemometric workflow used in the study combining: (1) chemometric approaches using NMR and mass spectrometry and (2) cell biological approaches using an osteoclast cytochemical marker (TRAP); Figure S2: Semi-prep-SFC chromatograms showing the monitoring (ELSD in red, UV 275 nmin green and UV220 in violet) and isolation windows of two potential bioactive substances (purple and yellow); Figure S3: Schematic representation of the experimental design to study *A. indica* fruit extract, microfractions and compounds.

## Sample Availability

Not available.
