# Supplementary material for: Biochemometry-Based Discovery of Phenylpropanoids from Azadirachta indica Fruits as Inhibitors of In Vitro Osteoclast Formation
Source: Molecules. 2022 Jun 4;27(11):3611. doi: 10.3390/molecules27113611 (PMC9182001; doi:10.3390/molecules27113611)
Supplement: Supplementary file 1 [file molecules-27-03611-s001.zip › molecules-1733544-supplementary.pdf]

Supporting Information

to

Biochemometry-based discovery of phenylpropanoids from *Azadirachta indica* fruits as *in vitro* inhibitors of osteoclast formation

Ammar Tahir[1], Carina Kampleitner[2], Theresa Wirglauer2, Ulrike Grienke[1], Oskar Hoffmann[2], Judith M. Rollinger[1*]

[1] Division of Pharmacognosy, Department of Pharmaceutical Sciences, University of Vienna, Josef-Holaubek-Platz 2, 1090 Vienna, Austria

[2] Division of Pharmacology and Toxicology, Department of Pharmaceutical Sciences, University of Vienna, Josef-Holaubek-Platz 2, 1090 Vienna, Austria

***Correspondence:** Judith M. Rollinger, e-mail: judith.rollinger@univie.ac.at

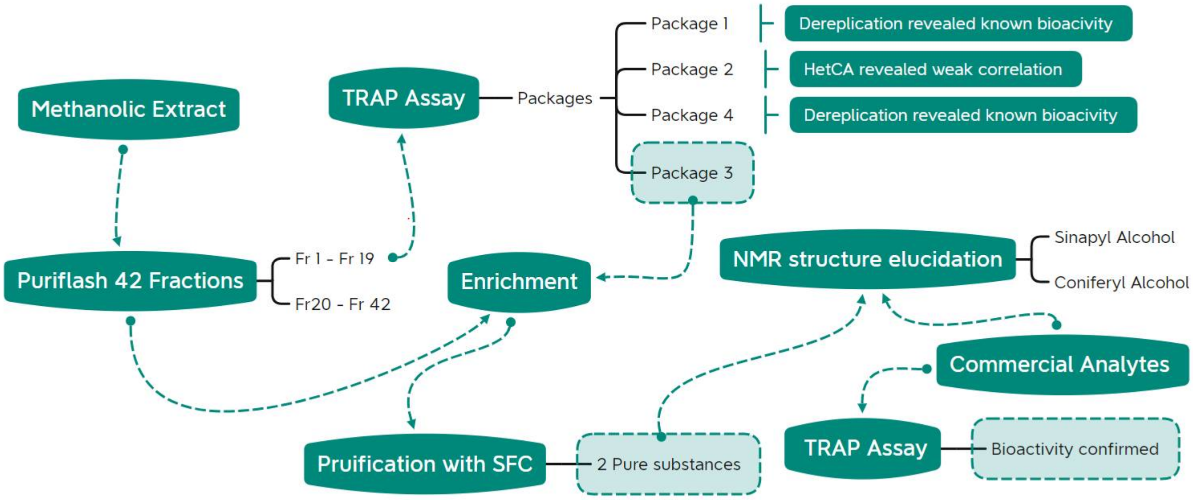

Figure S1 Biochemometric workflow used in the study combining: 1) chemometric approaches using NMR and mass spectrometry and 2) Cell biological approaches using an osteoclast cytochemical marker (TRAP).

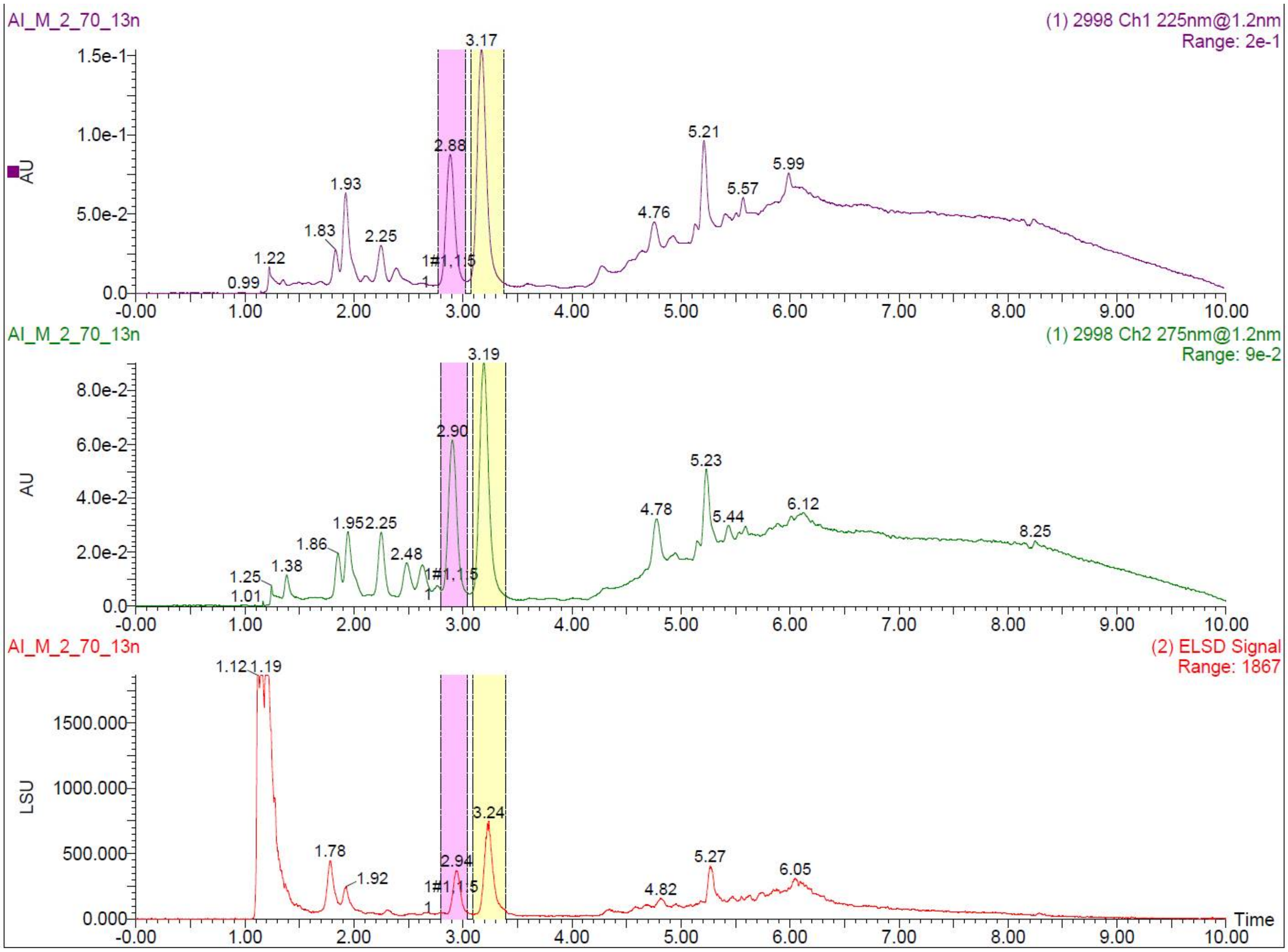

Figure S2 Prep-SFC chromatograms showing the monitoring (ELSD in red, UV 275nmin green and UV220 in violet) and isolation windows of two potential bioactive substances (purple and yellow).

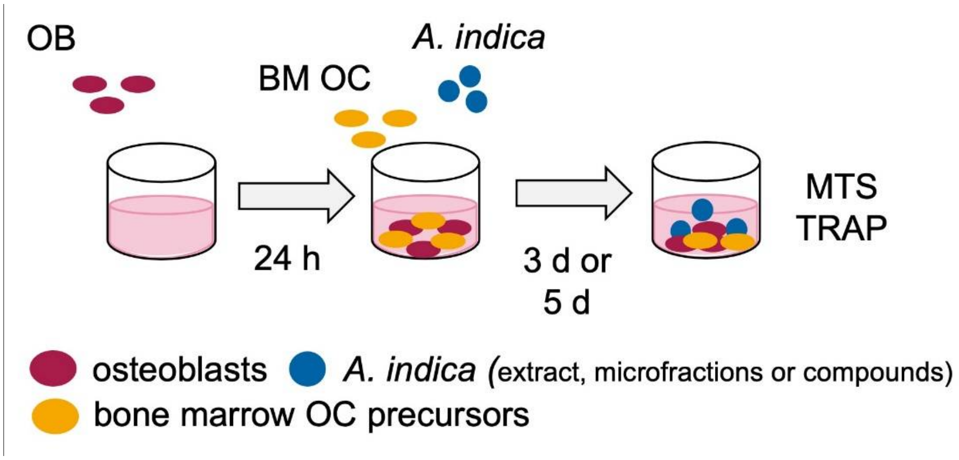

Figure S3: Schematic representation of the experimental design to study A. indica fruit extract, microfractions and compounds.
